# Biobanks in Horizon 2020: sustainability and attractive perspectives

**DOI:** 10.1007/s13167-018-0153-7

**Published:** 2018-11-23

**Authors:** Judita Kinkorová, Ondřej Topolčan

**Affiliations:** Department of Immunochemistry, University Hospital Pilsen, Edvarda Benese 1128/13, 305 99 Pilsen, CZ Czech Republic

**Keywords:** Predictive preventive personalised medicine, Biobank, Horizon 2020, Research infrastructures, Sustainability, Roadmap, Priority, European Commission, Excellence, Teaming, Coordination, Social, Ethic, Legal, Economic, Education, Innovation, Omics, Public private partnership, Network, IT, Digital services, Cross-disciplinary, Patient, Big data, Well-being, Society

## Abstract

Biobanks have during last two decades gained an important role in the whole process of biomedical research and health care not only in Europe but also worldwide. Biobanks are one of the pillars in personalised medicine tackling all its aspects such as prevention, diagnosis, treatment and monitoring closely the specific characteristics of an individual patient. The current and future power of biobanks is the amount of samples of high-quality and related information available for current and future research of diseases, for optimising patients´ prevention, diagnosis, treatment and monitoring. The material stored in biobanks is a treasure for future technologies that will be able to utilise the currently uncovered information and knowledge. A great and growing number of samples and related information also opens new ways in how to tackle the big data problems and population studies. Biobanks play a substantial role in drug discovery, development and validation. Biobanks are not only an issue of biomedical research, but are becoming a public issue involving patients, to actively participate in biobanking with respect to ethical, legal and social issues. And, finally, biobanking as a multidisciplinary and modern field of science requires appropriate education at all levels of society. To be a world leader in the field of biobanking requires wide international and interdisciplinary collaboration. The topic-dedicated programmes released by the European Commission sustainably support biobank development in Europe and the main tool is the biggest European Union (EU) research and innovation programme ever—Horizon 2020. This article reviews the main Horizon 2020 biobanking projects, financing schemes and the future perspectives.

## Introduction

Framework programmes (FPs) of the European Union (EU) are the biggest European research and innovation programmes with the aims to support the creation of the European Research Area (ERA); the 6th framework programme (FP6, 2002–2006) and, later, the 7th framework programme (FP7, 2007–2013) were key tools to respond to Europe’s needs in terms of jobs and competitiveness, and to maintain leadership in the global knowledge economy. Currently, the 8th framework programme (FP8, 2014–2020), called Horizon 2020, aims to support research, development and innovation in all scientific branches to make Europe a word leader. Horizon 2020 (FP8) is the biggest EU research programme ever with nearly €80 billion of funding available over 7 years, in addition to the private investment that this money will attract [1]. The programme supports excellent science, competitive industry and tackling societal challenges (SCs), including "Societal Challenge 1" (SC1) focused on health, demographic change and well-being, and is devoted to the wide range of topics and challenges in biomedical research and human health with a total budget of € 7.472 billion [2].

**Table:** Structure of Horizon 2020

Excellence Science	Industrial Leadership	Societal Challenge (SC)
European Research Council	ICT	Health, demographic change and wellbeing (SC1)
Future and Emerging Technologies	Nanotechnologies, materials, biotechnologies, manufacturing	Food security, sustainable agriculture, marine and maritime research, and the bio-economy
Marie Skłodowska Curie Actions	Space	Secure, clean and efficient energy
Research Infrastructures (including e-infrastructure)	Access to risk finance	Smart, green and integrated transport
	Innovation in SMEs	Climate action, resource efficiency and raw materials
		Europe in a changing world-inclusive innovative, reflective societies
		Secure societies

Spreading excellence and widening participation is the fourth section of Horizon 2020, new in framework programmes, enabling fast and efficient exchange of knowlege, experience, talents and enabling wider participation of countries with low participation rate. This section consists of three main actions: teaming, twinning and Era chairs. 

In the framework programmes, a lot of financing schemes, research and innovation actions and different activities are supported. Special topics and challenges require special project organisation and schemes.

Special research requirements, retained fragmentation of research in Europe, great research potential of European institutions, research centres, academia, industry and other aspects have changed the research environment and require new approaches and new solutions for biomedical research in Europe.

One of new phenomena and the key word in the last framework programmes (FP6 and FP7) was biobank or biobanking as an integral part of personalised medicine approaches to the human health and health care systems. In Horizon 2020, in some topics of SC1, biobank involvement is required [four topics in work programme (WP) SC1 2018–2020], and collaboration with registries, repositories and research infrastructures is recommended [3].

Personalised medicine is an excellent example of new innovative approaches, collaboration across disciplines and multidisciplinarity. Biobanking as a dramatically developed discipline is an integral part of personalised medicine.

Biobanking is multidisciplinary because scientific practice has become increasingly interdisciplinary, with rapid information and flexible and dynamic research collaborations around the world [4]. Research projects are frequently global in nature, involving teams with different types of expertise, such as clinicians, laboratory staff and researchers, bioinformaticians, statisticians and other data analysts [5].

## Material and methods

This study was based on the following WPs: Horizon 2020 WP 2018–2020 8. Health, demographic change and well-being [3], 4. European research infrastructures (including e-infrastructures) [6–8], 3. Marie Skłodowska-Curie Actions [9–11], 15. Spreading Excellence and Widening Participation [12–14], WPs of BBMRI-ERIC: WP 2016, WP 2017, WP 2018 [15–17], and following databases e-CORDA- External Communication Research Data Warehouse [18], and Participant Portal [19].

## Overview and discussion

### Biobanking is an EU priority

The EU considers its member states as world leaders in the development of biobanking infrastructure to support research, making huge investments each year to support such initiatives [5]. Not only one institution, nor one member state is able to solve all the problems and answer all questions regarding biobanking. Biobanks are embedded in complex networks of research collaborations that span regions, countries and the globe [4]. Biobank development has been stated as one of the key challenges in the last two decades in EU and the approach how to support the development and functioning of biobanks in Europe was one of key factors in FP7 and subsequently in Horizon 2020.

### Biomedical infrastructures

Research infrastructures are one of the most important EU tools to identify and solve the most actual challenges in European research. They provide facilities, resources, services and equipment to widely support and accelerate the whole process. Biobanks as one of the most important instruments are represented by collections, archives and databases. Research infrastructures can be concentrated on a single spot, distributed or virtual [20]. Since 2002, the European Strategy Forum on Research Infrastructures (ESFRI) identified needs for research infrastructures in Europe in the main branches of current research and formulated a strategic document, the ESFRI Roadmap, defining the specific research infrastructures essential to foster European research and economy [21]. The ESFRI Roadmap was first published in 2006 and, based on that, 35 projects were identified in all the scientific branches [22]. Three main biomedical grouped infrastructures were established: the Biobanking and Biomolecular Resources Research Infrastructure (BBMRI-ERIC), the European Advanced Translational Research Infrastructure in Medicine (EATRIS), and the European Clinical Research Infrastructures Network (ECRIN).

BBMRI-ERIC was prioritised in the roadmap of the ESFRI roadmap, and for the preparatory phase (2008–2011), BBMRI was funded from the FP7 [23]. Since 2013 when BBMRI was officially awarded the community legal framework for a European Research Infrastructure Consortium (ERIC), BBMRI has been the leading research and development body in biobanking. BBMRI-ERIC aims to establish, operate and develop a pan-European distributed research infrastructure in order to facilitate access to biological resources and associated data as well as facilities and to support high-quality biomolecular and biomedical research. BBMRI-ERIC can participate in Horizon 2020 projects as partner or coordinator.

### Horizon 2020: types of projects and funding schemes

Research infrastructures are very specific and innovative funding schemes. Horizon 2020, as the biggest EU research programme, offers many other types of “grants” (funding schemes) depending on the type of research, type of collaboration and other specificities. The financial tools for basic and applied research are research and innovation actions (RIAs), activities aiming at creating new knowledge and to explore the feasibility of a new or improved technology, product, process, service or solution. Beside basic and applied research, technology development and integration, testing and validation on a small-scale prototype in a laboratory are supported. Projects may contain closely connected but limited demonstration or pilot activities aiming to show technical feasibility in a near-to-operational environment. The EU funding rate is 100% [1].

### Research and innovation action “biobank” projects in Horizon 2020

DOLORisk: the aim is to understand neuropathic pain pathophysiology as a potential health burden in the future. The identification of risk factors and protective mechanisms ranging from molecular pathways to societal impacts will be identified during the project duration. A result of the project is to create a platform for improving diagnosis of neuropathic pain and to stratify patients according to risk profile, employ preventive strategies and finally to develop novel therapeutics [24].

LifeBrain: the aim of the project is to identify determinants of brain, cognitive and mental health during the whole human life, namely at different stages of life. To achieve the goal, integration and harmonisation of main neuroimaging studies of different age subgroups and changes in European population will be provided. The project will, as a result, create a database of fine-grained brain, cognitive and mental health measures of a group more than 6000 individuals [25].

EGI-Engage: a project of 43 partners with a mission to accelerate the implementation of the Open Science Commons vision. EGI-Engage brought together researchers from different fields of research to create easy and open access to the innovative digital services, storage, data, knowledge, communication and expertise complementing community-specific capabilities. The Open Science Commons is based on three main parts: the e-Infrastructure Commons; the Open Data Commons, for access, use and reuse data; and the Knowledge Commons [26].

PhenoMeNal: The project will develop and distribute e-infrastructure for the processing, analysis and information-mining of the massive amount of phenotyping and genotyping data and medical molecular information. This infrastructure will be integrated, secure, permanent, on-demand, service-driven, privacy-compliant and sustainable [27].

ALEC: The project studies the effects of factors that cause poor lung function, respiratory disability and the development of chronic obstructive pulmonary disease (COPD). Lung growth and lung functions will be examined with the use of existing cohorts that cover the whole life, and which have followed the respiratory health status of over 25,000 European children and adults from the early 1990s up to the present [28].

LIFECYCLE: The project will establish the EuroCHILD Cohort Network. The network will be based on existing, successful pregnant cohorts, child cohorts and biobanks, and a governance structure with respect to the national and European ethical, legal and societal issues will be developed. At the same time, a shared data-management platform and data-harmonisation strategies will be built. LIFECYCLE's aim is to enhance the EuroCHILD Cohort Network with the use of new integrated data on early life stressors related to socio-economic, migration, urban environment and lifestyle determinants. As a result, hypothesis-driven research on early life stressors influencing cardio-metabolic, respiratory and mental health trajectories during the full lifecycle and the underlying epigenetic mechanisms will be performed [29].

CORBEL: Ageing populations and chronic disease are the big problem not only in Europe. The problem of ageing population supports the acceleration of translation of biomedical discoveries to new, innovative and cost-effective treatments. The ESFRI Biological and Medical Research Infrastructures (BMS RI) follow every step in this process; joining scientific capabilities and sharing services will transform the understanding of biological mechanisms and accelerate its translation into modern and innovative medical care. CORBEL aims to develop services, tools and data management necessary for cutting-edge European research and innovative projects: all together, the BMS RIs will establish a sustainable foundation services for collaborative biomedical research in Europe. Combined infrastructure capabilities will be embedded into the scientific workflow of all advanced users [30].

EYE-RISK: Age-related macular degeneration (AMD) is one of the world’s most important age-related blinding disorders. In the project, the use of epidemiological data describing lifestyle, nutrition, molecular genetics, clinical phenotype and retinal imaging related to existing longitudinal European epidemiological cohorts and biobanks facilitate the global insights, which are necessary for long-lasting prevention and therapy for AMD [31].

UM Cure 2020: The UM Cure 2020 project is focused on the treatment of uveal melanoma (UM) metastases. The aim is to identify and validate the preclinical therapeutic approaches. That is why the consortium brings together UM experts in patient care and basic, translational and clinical research, and also patient association representatives. To move from patient tissue characterisation to preclinical evaluation of a single drug or combinations of drugs, and to identify new more efficient treatment, an ambitious multidisciplinary approach is proposed [32].

MOODSTRATIFICATION: Consists of a consortium of 11 partners of different specialisations (psychiatrists, immunologists, epidemiologists and industry) from 7 countries. For stratifying groups of patients with a major depressive episode, leukocyte immune profiles will be used for the first time. The involvement of national and international patient associations will facilitate the research. This will be the first lab-based therapy stratification in psychiatry made ready for the clinic [33].

MESI-STRAT: Breast cancer (BC) is a severe and complex disease with very high prevalence in the EU. Great proportions of the tumours are oestrogen receptor-positive (ER+), and are treated with endocrine therapies (ETs). The aim of MESI-STRAT is to develop new concepts for knowledge-based stratification of patients into subgroups based on different ET resistance mechanisms. Expected results will be predictive models for (1) patient stratification prior and during ET; (2) recurrence risk assessment when ending ET; (3) marker panel development to guide targeted therapies for ET-resistant patients; and (4) novel ET resistance mechanism-based therapy design [34].

EDIReX: The goal of the EDIReX project is to establish a European infrastructure which offers trans-national access (TA) of patient-derived tumour xenograph (PDX) resources to researchers both from academic and industrial cancer sectors. It will also include the distribution of cryopreserved samples for research to third parties, and models the structured modern biobanks, and the performance of efficacy studies [35].

RESSTORE: The subject of the project is stroke, the second leading cause of death in the world. RESSTORE will direct the clinical assessment of regenerative cell therapy based on emerging preclinical and pilot clinical evidences. The aim of the project is to improve stroke recovery and patient quality of life. RESSTORE will for the first time explore functional recovery and safety of intravenous infusion of allogenic adipose tissue-derived mesenchymal stem cells (ADMSCs) in a group of 400 stroke patients. The project will spread over the full value chain in the field (large-scale Good Manufacturing Practices (GMP) cell production, biomarker discovery, clinical testing and understanding of the restoring mechanisms, biobanking, modelling, economic studies, exploitation and a communication plan) and will offer new advanced therapies for European patients with brain diseases [36].

AARC2: The project “Authentication and Authorisation for Research and Collaboration 2” builds upon the previous project AARC1. The aim is to design an authentication and authorisation infrastructure (AAI) framework that enable researchers from various biomedical fields to access the whole research and infrastructure service portfolio with one login, and to meet their current needs in international collaboration. AARC2 will provide guidelines and a policy toolkit for research collaborations, guidelines covering assurance, General Data Protection Regulation (GDPR) considerations for research infrastructures and security aspects. AARC2 will map researchers' requirements to concrete service offerings, to support research (e-infrastructures) to implement the integrated architecture and policy frameworks developed by the AARC project, to offer different trainings, and to enhance the integrated architecture [37].

EOSCpilot: The EOSCpilot project will support the first phase in the development of the European Open Science Cloud (EOSC) as described in the EC Communication on European Cloud Initiatives in 2016. The EOSCpilot project will make possible reuse of data resources and will provide an important step towards building a dependable open-data research environment. It means that data from publicly funded research is always open and there are clear incentives and rewards for the sharing of data and resources [38].

The ADOPT BBMRI-ERIC project in the H2020, RIA scheme is specific and closely related to the activities of BBMRI-ERIC with the aim to boost and accelerate implementation of BBMTI-ERIC and its services. BBMRI-ERIC thus will provide access to the collections of the European research community, expertise and services building on the results and outcome of ADOPT BBMRI-ERIC [39].

The objectives of them are the most important or most frequent diseases in the European population, e.g. brain research and neurodegenerative processes and mental health connected with ageing population (Lifebrain, MOODSTRATIFICATION), chronic disease and ageing population (CORBEL), neuropathic pain (DOLORisk), age-related macular degeneration (EYE-RISK), respiratory disability (ALEC), stroke (RESSTORE), cancer (EDIReX) and so on.

All the mentioned projects cover a wide range of biomedical and related topics and use a wide range of scientific approaches and methods, e.g. modern-omics techniques and technologies, imaging, new biomarker discovery, validation and use, patient stratification, cohort studies, risk and environmental factors, a wide range of social aspects, modelling and algorithms, new strategies and policies, biostatistics, ICT, big data, drug development, new therapy identification, and innovative and more effective treatment.

Research activities include basic, experimental, translational, clinical, applied, interdisciplinary and cross-disciplinary research. Disease-oriented research projects are designed to include all aspects of the health care process from prevention, prediction, early diagnosis, optimal treatment and follow up, rehabilitation and others by means of personalised medicine principles and approaches. Widely designed projects require specifically assembled consortia getting together partners from academia and industry, the pharmacy sector, policy makers, patient organisations, small- and medium-size enterprises (SMEs), businesses, civil society organisations, education establishments, science & society centres and others.

Dissemination and promotion of results, learning and education modules or courses are integral parts of the projects.

### Coordination and support action “biobank” projects in Horizon 2020

The second most frequent project type in Horizon 2020 is coordination and support action (CSA), accompanying measures such as standardisation, dissemination, awareness-raising and communication, networking, coordination or support services, policy dialogues and mutual learning exercises and studies [1]. Other actions will be mentioned with the project.

Projects can be submitted as one stage if it means a full proposal by the given deadline, or two stages. The consortium consists of the coordinator and other partners; the number of partners depends on the project topic and the type of action.

SPIDIA4P is a typical CSA project following SPIDIA, which was a 4.5-year project funded in the FP7 programme. The project meets the needs of the standardisation and improvement of pre-analytical procedures for in vitro diagnostics. A lot of new pre-analytical technologies were developed. Within the CEN/technical committee 140 for “In vitro medical devices”, SPIDIA’s results enabled developing and introducing the first nine CEN technical specifications (CEN/TS). The SPIDIA4P project builds on SPIDIA’s results and is funded by the Horizon 2020 research and innovation programme. The consortium consists of 19 experienced partners from a variety of sectors such as private industry including SMEs, public institutions and one European standards organisation. Because of great success, the project is again coordinated by QIAGEN GmbH. It plans to initiate, develop and implement a comprehensive portfolio of an additional 14 pan-European pre-analytical CEN/TS and ISO/IS documents as well as external quality assessment schemes (EQAs), addressing the important pre-analytical workflows applied to personalised medicine [40].

RItrain: Currently, about 5O research infrastructure (RI) work exist or are in development in Europe. The great number of RIs requires tools, beside others, to develop a flagship training programme enabling RIs across all domains to gain expertise on governance, organisation, financial and staff management, funding, Intelectual Property (IP), service provision and outreach in an international context. To reach this goal, it is necessary to assemble experts who have set up and managed RIs from concept to maturity [41].

The main question of the project Genetics Clinic of the Future (GCOF) CSA is if the clinical implementation of genomic technologies is relevant and responsive to the needs of all. A variety of experts as clinical geneticists, genomics researchers, bioinformaticians, social scientists, ethicists and patient representatives has to work closely together to develop basic parts of the genetics clinic: data sharing and control, informed consent, patient and citizen involvement and the role of genome data within and beyond the clinic. The different disciplines cannot work in parallel, but have to be collectively involved from the problem definition to the design of solutions [42].

The B3Africa CSA aims to contribute to improvement of health and health care in the population of Africa. One step is to implement a cooperation platform and technical informatics framework for biobank integration between Africa and Europe. The collaboration will facilitate harmonisation of the ethical and legal issues, biobank data sharing and knowledge sharing between biobanks and researchers from both continents. The two main partners from the relevant initiatives, including the Human Heredity and Health in Africa project (H3Africa), the European BBMRI-ERIC, the LMIC Biobank and the Cohort Network (BCNet), guarantee strategic international cooperation [43].

### Teaming and twinning in Horizon 2020

In the structure of Horizon 2020, there is a new phenomenon, beside the three pillars (excellent science, industrial leadership, and societal challenges), the specific objective of “Spreading Excellence & Widening Participation” which includes “teaming” excellent research institutions with lower-performing counterparts to create or upgrade centres of excellence, and “twinning” institutions, including staff exchanges, expert visits and training courses [1].

The CY-Biobank project (funded under "Teaming of excellent research institutions and low performing Research, Development and Innovation (RDI) regions") is based on Cypriot population investigations of diseases and eHealth as priorities of the "Smart Specialisation Strategy" of the Cyprus Government that serve for creating a Centre of Excellence (CoE) with two spear heads: biobank and a research facility for developing the Cyprus Human Genome Project [44].

Project ePerMed funded under Spreading Excellence illustrates the experience sharing. The International Consortium for Personalised Medicine (ICPerMed) contributes to the implementation of personalised medicine principles in prevention and treatment into the Estonian health care system. The main objectives of the project are to exchange know-how and experience, to improve the connections between clinicians and genomic researchers, to increase soft skills, and to increase the visibility of scientific excellence and its potential [45].

Project CETOCOEN Excellence (funded under "Teaming of excellent research institutions and low performing RDI regions") is directed to exploit research capacities built in Central Europe with a support of the European Structural and Investment Funds to address major scientific and societal challenges. The project is funded under "Teaming of excellent research institutions and low performing RDI regions" [46].

### Marie Skłodowska-Curie action “biobank” projects in Horizon 2020

Project proposals can be submitted as Marie Skłodowska-Curie action, e.g. Marie Skłodowska-Curie Innovative Training Networks (MCSA-INT-ETN), or Marie Skłodowska-Curie Individual Fellowship MCSA-IF-EF). Marie Skłodowska-Curie action is a part of the first pillar of Horizon 2020: excellent science [1].

The idea is to support young scientists in different ways to offer participation in solving of specific scientific tasks: dry storing as an alternative biobanking strategy in the frame of the DRYSTORE project [47], or the establishment of national and European regulation on umbilical cord blood (UCB) biobanking in the REGUCB project [48]. Creating innovative European PhD training network in bone pain was the topic for the BonePain project [49]. The TRAIN project [50] focuses on a multidisciplinary research training programme on obesity-related diseases. The RISTRAD project [51] deals with translational study combining genome-wide association studies of patients with cardiac arrhythmia for novel genes uncovered. The project DesignerAntibiotics aims to prevent aminoglycoside antibiotic-related deafness [52].

A special funding scheme in Marie Skłodowska Curie action is research and innovation staff exchange (RISE). The project DRYNET is one of those, merging the partner’s expertise, theoretical/biophysical/mathematical modelling, cellular/molecular/insect biology, embryology and mechanical engineering into a coherent approach towards dry storage of cells/germplasm. DRYNET strengthens the network between different disciplines and boosts European competitiveness in biobanking [53].

### Other “biobank” projects in Horizon 2020

The European Commission (EC) is as a partner involved in the public/private partnership (PPP) Innovative Medicine Initiative (IMI1, 2008–2013) [54] together with the European Federation of Pharmaceutical Industry and Associations (EFPIA) [55] funding health research and innovation. The IMI facilitates open collaboration in research to advance the development of and accelerate patient access to personalised medicines for the health and well-being of all, especially in areas of unmet medical need.

Innovative Medicine Initiative 2 (IMI2, 2014–2020), continues in previous interest in biobanking in two RIA projects: INNODIA focused on translational approaches to disease modifying therapy of type 1 diabetes (T1D) with the aim to achieve a break-through in the way in which the study of T1D unable to move closer towards prevention and cure of T1D. The project consortium consists of coordinator and 33 partners [56]. RESCUE project (Respiratory Syncytial virus RSV Consortium in Europe) aims to develop robust evidence on RSV disease burden and economic impact, create multidisciplinary and multi-stakeholder community to perform future pivotal trials for RSV vaccines and therapeutics, and thus to contribute to improvement health and well-being in Europe [57].

The SME instrument is a special EC tool to support highly innovative SMEs. The project SPARK-TUBE, funded under both the SME instrument, "Mainstreaming SME support", especially through a dedicated instrument and also the SC1 of the pillar of societal challenges: health, demographic change and well-being, was invented to make current complex methods of blood preservation and biobanking more efficient, robust and simplified, and is expected to become a universal biobanking platform [58].

In addition to the above-mentioned projects, BBMRI-ERIC publishes every year a WP with an actualized list of running projects with BBMRI-ERIC participation. In 2016, 7 projects were running: ADOPT BBMRI-ERIC, B3Africa, RItrain, CY-Biobank, PhenoMeNal, EGI-Engage and CORBEL. In 2017, the following projects were introduced: AARC2, DRYNET, EOSCpilot and SPIDIA4P, and, finally, in 2018, three new projects started: EDIReX, EOSC hub [59] and ID-EPTRI [60], the last two commencing January 1, 2018.

In the last decade, several international initiatives have emerged to coordinate and harmonise biobanking [61]. The aim of these activities is their wide international extension and infrastructural nature, based on the progress in all the fields of biomedical research. A good international example is the Canadian-led Public Population Project on Genomics and Society P3G [62].

## Discussion

The EC recognised the importance of international biobanks and has funded many activities, projects and infrastructures that act in the biobank environment. Infrastructures (including e-infrastructures) are a special important tool of the EC to ensure access to world-class facilities (H2020, WP 2018–2020). The number and variability of biobank projects supported by the EC in the frame of H2020 documents the overall importance of biobanking in biomedical research in Europe. Many of the current projects follow from previous project results, develop and/or expand them and thus accelerate the processes in biomedical research in Europe.

Regarding the future perspectives, fragmentation is still indicated as the problem that slows down and reduces the process of scientific discovery and innovation. Biobanks are a tool to accelerate scientific discovery, but it will be crucial to improve their quality, interoperability and sustainability.

Europe currently boasts valuable and patent cohorts, and the future development is based on a solid basis requiring modern and innovative approaches to utilise these advantages with respect to the Ethical, Social and Legal Issues (ESLI). ESLI issues, the openness of biobanks for samples sharing, and personal information protection [currently the EU General Data Protection Regulation (GDPR) and The European Code of Conduct for Research Integrity] are today's main objectives of the "science" of biobanking.

Biobanking in Europe has moved forward from the initiating phase to build real biobanks as organised collections consisting of biological samples and associated data for research and personalised medicine, supporting networking between biobanks, other stakeholders, and globally [63].

The leader in biobanking in Europe is BBMRI-ERIC, publishing WPs every year with main goals identified for the period. In WP 2018, the main goal is to build and strengthen value-added, sustainable biobanking. This ambitious goal can be achieved if the BBMRI-ERIC team, the national nodes and the various biobanks join efforts [64].

## Conclusion and expert recommendations

Biobanks are collections of biological samples (here, human biological samples) and associated data. This unique combination of dissimilar sets makes biobanks very special regarding their position in biomedical research, in ethical requirements, multidisciplinarity and international collaboration. Biobanking was mentioned as one of the "ten ideas changing the World right now“ in 2009 in Time magazine [65]. Since 2000, biobanks have become a pillar of personalised medicine and their role has been increasing. Biobanking has become a science involving a lot of professions and specialisations such as: biomedical researchers, bioinformatics, IT specialists, lawyers, and also industry and patients´ organisations, and others. Biobanks are closely related to research of new biomarkers, drugs [66] and personalised treatment regimes [67].

The greatest power of biobanks is the large number of stored samples of special diseases, and special patient cohorts, for retrospective or prospective studies. The future value of biobanks is at the moment insufficiently known, because in the next decades new research methods, approaches and technological achievements will be discovered and there will be enough material for new discoveries.

Until now, there have been three international biobanking leading "bodies“, ISBER, ESBB and BBMRI-ERIC, governing the biobanking. The International Society for Biological and Environmental Repositories (ISBER) is a worldwide biobanking organisation which creates platforms and opportunities for wide international collaboration, networking, harmonisation, education and innovations for biological and environmental repositories. ISBER supports cooperation, facilitates education and training opportunities and demonstrates international state-of-the-art policies, processes and research findings as well as innovative technologies, products and services [68]. The mission of the European, Middle Eastern and African Society for Biobanking (ESBB) is to advance the field of biobanking and to support the research focused on healthcare, agriculture and the environment [69]. And, finally, the biggest biomedical infrastructure for biobanking in Europe, BBMRI-ERIC, brings together all the main players from the biobanking field—researchers, biobankers, industry and patients—to boost biomedical research. BBMRI-ERIC has several innovative tools to offer high-quality management services, ethical, legal and societal issues, and a lot of online tools and software solutions. The vision of BBMRI-ERIC is to make new treatments possible [23].

To summarise, for the future in biobanks and biobanking, a multidisciplinary approach is necessary, international collaboration is essential [70] and education and research programmes are the basic prerequisites for “healthy and personalised” development of biobanks.
